# In Silico Design of Novel EpCAM-Binding Aptamers for Targeted Delivery of RNA Therapeutics

**DOI:** 10.3390/nano14211727

**Published:** 2024-10-29

**Authors:** Julia Driscoll, Piyush Gondaliya, Abbye Ziemer, Irene K. Yan, Yash Gupta, Tushar Patel

**Affiliations:** 1 Department of Transplantation, Mayo Clinic Florida, 4500 San Pablo Road, Jacksonville, FL 32224, USAyan.irene@mayo.edu (I.K.Y.); 2 Division of Infectious Diseases, Mayo Clinic Florida, 4500 San Pablo Road, Jacksonville, FL 32224, USA

**Keywords:** aptamers, molecular dynamics, cholangiocarcinoma, epithelial cell adhesion molecule, nanovesicle

## Abstract

Aptamers are short DNA or RNA sequences that adopt 3D structures and can bind to protein targets with high binding affinity and specificity. Aptamers exhibit excellent tissue penetration, are inexpensive to produce, and can be internalized by cells. Therefore, aptamers are attractive targeting ligands to direct the delivery of theranostic agents to the desired cells. Epithelial cell adhesion molecule (EpCAM) is a tumor-associated antigen that is aberrantly overexpressed on many epithelial-derived cancers, including on cholangiocarcinoma (CCA) cells. Its expression on treatment-resistant cancer stem cells, along with its abundance in the CCA tumor microenvironment, highlights the need to develop EpCAM-targeted therapies for CCA. Herein, an *in silico* approach was used to design and screen DNA aptamers capable of binding to the EpCAM monomer and homodimer. Two aptamers, PLD01 and PLD02, met the selection criteria and were validated in vitro. Both aptamers exhibited high affinity for EpCAM+ CCA cells, with negligible binding to EpCAM- leukemia cells. Modified versions of PLD01 and PLD02 were successfully incorporated into the membranes of milk-derived nanovesicles. PLD01-functionalized nanovesicles enabled EpCAM-targeted delivery of the therapeutic cargo to CCA cells. In summary, these EpCAM-targeting aptamers can be utilized to direct the delivery of theranostic agents to EpCAM-expressing cells.

## 1. Introduction

Aptamers, or chemical antibodies, are short (<100 bases) oligonucleotide sequences composed of single-stranded DNA or RNA that adopt tertiary structures with diverse shapes. Their three-dimensional structural conformations enable them to bind with high affinity to specific protein targets through shape recognition. Consequently, aptamers can be used as molecular probes for targeted imaging or therapeutic applications [1,2]. Aptamers offer many advantages over the more widely used and studied antibodies. Namely, they are simpler and inexpensive to synthesize, have short production times, exhibit minimal batch-to-batch variation, are stable when stored, possess superior solid tumor penetration, and have a wider range of potential targets, including non-immunogenic epitopes [3].

The use of aptamers for selective delivery of therapeutic cargo to target cells of interest is garnering interest as an alternative to the use of conventional antibodies [4]. The anti-tumor effects of aptamer-directed therapies have been reported in several pre-clinical studies. Several aptamers have been tested in early phase clinical trials and have been shown to be well-tolerated and also to synergize with combination treatment [5,6].

Epithelial cell adhesion molecule (EpCAM) is an attractive candidate for aptamer-targeted therapeutics. EpCAM is a type 1 transmembrane glycoprotein that is expressed on embryonic stem cells, hepatic progenitor cells, and healthy epithelial tissues and furthermore is aberrantly overexpressed in many epithelial-derived cancers [7,8,9,10,11]. EpCAM has been shown to contribute to tumor growth and progression through several mechanisms. The complexation of the EpCAM intracellular domain with members of the Wnt signaling pathway drives expression of oncogenes such as c-myc [12]. EpCAM is also expressed on cancer stem cells (CSCs), and can contribute to tumor invasion, metastasis and treatment resistance [10]. EpCAM expression levels are correlated with negative prognosis and treatment response in cholangiocarcinoma (CCA), breast, bladder, and ovarian cancers, amongst others [11,13,14]. Indeed, EpCAM has been targeted for delivery of nanotherapeutics and immunotherapies such as chimeric antigen receptor (CAR) T-cell therapy and antibodydrug conjugates [15,16].

Cholangiocarcinoma is often diagnosed at the advanced and unresectable stage [14]. EpCAM is therefore an attractive, tumor-associated antigen for tumor targeting of these cancers for which a targeted therapeutic approach is highly desirable. Given their ease of modification, aptamers that target EpCAM can be conjugated with various moieties to generate EpCAM-directed diagnostics or therapeutics. Therefore, we sought to design DNA aptamers that possess high affinity to and specificity for EpCAM using computational modeling. We evaluated the EpCAM targeting ability of these aptamers in EpCAM-expressing CCA cells and demonstrated their utility as targeting ligands to develop EpCAM-targeted therapeutic nanovesicles.

## 2. Materials and Methods

*Cell culture*. HL-60 promyeloblast leukemia cells and HEK293T cells were obtained from ATCC (ATCC, Manassas, VA, USA). HL-60 cells were grown in Iscove’s modified Dulbecco’s medium (Gibco, Billings, MT, USA) supplemented with 20% FBS. HEK293T cells were cultured in high-glucose Dulbecco’s modified eagle medium (DMEM-HG) (Cytiva, Wilmington, DE, USA) supplemented with 10% fetal bovine serum (FBS) (Gemini Bio Products, Sacramento, CA, USA) and 1% penicillin–streptomycin (P/S) (Thermo Fisher Scientific, Waltham, MA, USA). HuCCT1 cholangiocarcinoma cells were obtained and cultured as previously described on collagen-coated plates and maintained in DMEM-HG with 10% FBS and 1% P/S [17].

*In silico docking and molecular dynamics*. The crystal structures of the EpCAM ectodomain (EpEX) monomer (PDB ID: 6I07) and homodimer (PDB ID: 4mzv) were obtained from the RCSB protein data bank as PDB files of the biological assembly [18]. The EpEX monomer was cropped from the structure of the EpCAM homodimer in complex with a single-chain variable fragment (ScFv) (Schrödinger Release 2023-3, Maestro, Schrödinger, LLC, New York, NY, USA) [19]. The sequences of the following DNA aptamers that have been characterized as binding to EpCAM were obtained to be used as seed aptamers for the *in silico* studies: JYK-01, Seq.ID 63, Epp166, and SYL3C (Table 1) [20,21,22,23]. The 3D folded structures of the seed aptamers were generated using the 3dRNA/DNA server (Xiao Lab) [24]. A protein preparation wizard was used to resolve the 3D structures, and protein–nucleotide docking between the EpEX monomer and each folded aptamer was performed using the standard mode, 70,000 rotations, and 30 poses. The docked poses with the lowest PIPER scores were selected for molecular dynamics (MD) simulation studies. For the MD simulations, an orthorhombic system with a buffer space of 10 Å (10 × 10 × 10) was built with TIP3P solvent and 0.15 M NaCl, and sodium ions were added to neutralize the system. Two hundred-nanosecond MD simulations of the docked poses were performed under normal temperature and pressure conditions using Schrodinger Desmond (Schrödinger Release 2023-3). The model was relaxed prior to simulation. At the completion of MD, simulations interactions diagrams were generated, and the raw data were extracted. The root mean square deviation (RMSD) plots were constructed to evaluate the stability of the EpEX–aptamer complexes. The root mean square fluctuation (RMSF) data were used to identify the aptamer-contacted amino acid residues and to classify the interactions. Novel EpCAM binding DNA aptamers were designed based on the analysis of the interaction dynamics between the seed aptamers and the EpEX monomer. The design process included identifying the aptamer structural motifs needed for stable EpCAM binding/interactions and refining the aptamer sequences to reduce the sequence length and stabilize the aptamer structure. MD studies with the novel DNA aptamers were performed with both the monomer and homodimer EpEX structures, as the homodimer predominates in vivo [25].

*Aptamer selection and synthesis*. The aptamers that met the following criteria were selected for in vitro validation: (1) short sequence (x < 50 nucleotides), (2) forms the classical loop-stem 3D structure, (3) terminal nucleotides do not interact with EpEX, and (4) the EpEX–aptamer complex RMSD becomes stabilized during the MD simulation. For the in vitro studies, the DNA aptamers were synthesized with a 5′ biotin tag (B-aptamer) and several phosphorothioate-modified nucleotides. For development of EpCAM-targeted nanovesicles, aptamers were also synthesized with a 5′-Cy5 tag and a 3′-triethylene glycol cholesteryl (TEGChol) tag (GenScript, Piscataway, NJ, USA).

*Serum stability assay*. Fifty micromolar of B-aptamers were incubated with 10% FBS for up to 24 h at 37 °C. A sample of plain B-aptamer preserved at −20 °C was also included. At selected time points (0, 3, 6, 9, or 24 h), samples were mixed with SmartGlow (Accuris Instruments, Edison, NJ, USA), resolved on a 3% agarose gel, and visualized using the SmartDock lightbox (Accuris Instruments).

*EpCAM expression*. Cell surface expression of EpCAM was evaluated on HuCCT1 CCA and HL-60 leukemia cells. Cells were stained with 1:50 EpCAM-AF647 (Cell Signaling, Danvers, MA, USA) or IgG1-AF647 isotype control antibody (R&D systems, Minneapolis, MN, USA) for 1 h on ice, then washed, and analyzed using NovoCyte flow cytometer (Agilent NovoCyte 2060, Santa Clara, CA, USA).

*Aptamer binding affinity*. Assays were performed with EpCAM-positive HuCCT1 or EpCAM-negative HL-60 cells. Endogenous biotin on HuCCT1 cells was first blocked by incubation with 0.01 mg/mL streptavidin (Fisher Scientific, Waltham, MA, USA) in blocking buffer (PBS supplemented with 0.2% NaN_3_ (Thermo Fisher Scientific), 2.5 mM MgCl_2_ (Millipore Sigma, Burlington, MA, USA), and 0.1 mg/mL yeast total RNA (Sigma)) for 30 min on ice and subsequently incubated with 0.03 mg/mL biotin (Millipore Sigma) for 30 min on ice. HL-60 cells were not subject to endogenous biotin blocking. The cells were then incubated with B-aptamers for 30 min on ice. The cells were washed and incubated with 5 µg/mL Alexa Fluor 647 conjugated streptavidin (Strep-AF647, Thermo Fisher Scientific) for 30 min on ice. Cells were then stained with viability dye (Live-or-dye 568/583, 1:1000, Biotium, San Francisco, CA, USA) for 15 min on ice. Thirty thousand events were acquired on the NovoCyte flow cytometer. Controls included the following: (1) cells stained with only Strep-AF647 and (2) a mixture of live and dead cells stained with viability dye. The data were analyzed in FlowJo (V10.8.1, Ashland, OR, USA), and the median fluorescence intensity (MFI) of the streptavidin-AF647 signal was used to evaluate aptamer binding affinity to the cells. One-site specific binding curves were constructed, and the dissociation constant (Kd) value of each aptamer was determined using GraphPad.

*Aptamer functionalization of nanovesicles*. Milk-derived nanovesicle (MNV) delivery vectors were isolated from fat-free milk as previously described [26]. MNVs (2 × 10^12^ particles) were stained with 5 μM Vybrant DiO (Thermo Fisher Scientific) and isolated by ultracentrifugation at 100,000× *g* for 30 min at 4 °C (Optima Max XP, Beckman Coulter, Brea, CA, USA). To develop aptamer-functionalized nanovesicles (T-MNVs), the DiO-stained MNVs (2 × 10^11^ particles) were resuspended in sterile PBS and mixed with 0.01, 0.1, or 1 μM Cy5-aptamer-TEGChol, and TE buffer was added to achieve a final volume of 100 μL. The MNVs were incubated at 37 °C for 90 min. The aptamer-functionalized MNVs were isolated by ultracentrifugation, then diluted to a final concentration of 3 × 10^8^ particles in 30 μL to prevent swarming. The aptamer decoration efficiency was assessed using imaging flow cytometry with an ImageStream (MK II, MilliporeSigma, Cytex Biosciences, Fremont, CA, USA) at 60× magnification, low flow speed, and with the *remove beads* feature disabled; 10,000 events of each sample were acquired. Single color controls included (1) DiO-stained MNVs, (2) unstained MNVs functionalized with 0.1 μM Cy5-aptamer-TEGChol, as well as a (3) dye only control consisting of 5 μM vibrant DiO in PBS that underwent ultracentrifugation and was resuspended in PBS. Data were analyzed using the IDEAS software (MilliporeSigma) and FlowJo.

*Nanovesicle uptake*. DiO-stained MNVs were functionalized with 0.1 μM or 1 μM of Cy5-PLD01-TEGChol as described above. HuCCT1 cells were treated with 2 × 10^11^ DiO-stained plain or aptamer-functionalized MNVs for 4 h, after which the cells were harvested and stained with viability dye. The data were acquired on the ImageStream system, and 30,000 events were acquired. Single color controls were also included. An adaptive erode mask was drawn around the cell perimeters, and an internalization feature was created for the green (MNV) detection channel that measures the ratio of the intensity of the green-stained MNVs inside the cell compared to the intensity in the entire cell as an MNV internalization score in the live, single, in-focus cell population.

*siRNA delivery*. Aptamer-functionalized MNVs were used for the delivery of siRNA. Five micromolar of siRNA against β-catenin (S100029743, Qiagen, Hilden, Germany) was prepared in OptiMEM (Gibco) in a total volume of 50 μL. Four microliters of Lipofectamine 2000 (Thermo Fisher Scientific) was prepared in OptiMEM, then equal volumes of the siRNA and lipofectamine solutions were mixed and incubated at room temperature for 15 min. MNVs (2 × 10^12^ particles) were added to the siRNA-lipofectamine solutions and incubated for 30 min at room temperature. The loaded MNVs (tMNVs) were isolated by ultracentrifugation and resuspended in 500 μL of sterile PBS. Therapeutic MNVs were functionalized with 1 μM Cy5-PLD01-TEGChol (T-tMNVs) or left unfunctionalized as controls (tMNVs). HuCCT1 cells were treated with 2 × 10^11^ tMNVs or PLD01 T-tMNVs (n = 3 biological replicates/treatment) for 48 h. Then, TRIzol (Thermo Fisher Scientific) was added, and RNA was extracted for further studies. Cells were transfected with 0.5 μM β-catenin siRNA as a positive control.

*RNA isolation and quantitative real time PCR (qRT-PCR)*. RNA was extracted using TRIzol, and the concentration was measured using a Nanodrop (Nanodrop 200c, Thermo Fisher Scientific). Three hundred nanograms of RNA was subjected to DNase digestion (RNase-free DNase set, Qiagen) and subsequently used for cDNA synthesis (iScript cDNA synthesis kit, Bio-Rad, Hercules, CA, USA). qRT-PCR was performed as follows: a master mix consisting of 2X SYBR green (Takara Bio Inc., Kasutasu Shiga, Japan), 5 µM β-catenin or U6 primer mix, and nuclease-free water was added to achieve a total volume of 8 µL/sample. The sequences of the primers used are as follows: β-catenin FWD, 5′- ATGGCCATGGAACCAGACAG-3′; β-catenin REV, 5′-TGGTAGTGGCACCAGAATGG-3′; U6 FWD, 5′-CTCGCTTCGGCCAGCACA-3′; and U6 REV, 5′-AACGCTTCACGAATTTGCGT-3′. Two microliters of (1:10 diluted) cDNA was combined with the master mix, and duplicates of each sample were included. The data were analyzed using the LightCycler software (96 SW 1.0, Roche Diagnostics, Mannheim, Germany), and β-catenin expression was normalized to that of the housekeeping gene. Data are reported as the average relative expression of β-catenin ± SD (n = 3 biological replicates).

*Statistical analysis*. Three independent experiments were performed for all in vitro experiments. The data were analyzed in GraphPad (GraphPad Software, La Jolla, CA, USA) and reported as mean ± SD (n = 3 biological replicates). One-way ANOVA analyses were performed to evaluate statistical significance.

## 3. Results

*Computational design of aptamer candidates*. Docking and molecular dynamics simulations of several seed DNA sequences that bind to EpCAM were performed to identify structural motifs and key sites of binding to the EpCAM extracellular domain (EpEX) monomer. Based on this, sixteen novel DNA aptamer sequences were designed. These were further screened *in silico* for binding to both the EpEX monomer and *cis* homodimer, as the *cis* homodimer predominates in vivo [25]. Simulations were performed for 200 ns, and the root mean square fluctuation data were used to identify the EpEX interacting residues. The root mean square deviation (RMSD) was used to evaluate the stability of the protein-aptamer complex.

A highly stable complex with EpEX was predicted on computational simulations for two unique DNA sequences, PLD01 and PLD02, and these were selected for in vitro validation. Their sequences are reported in Table 2.

**Figure 1:**
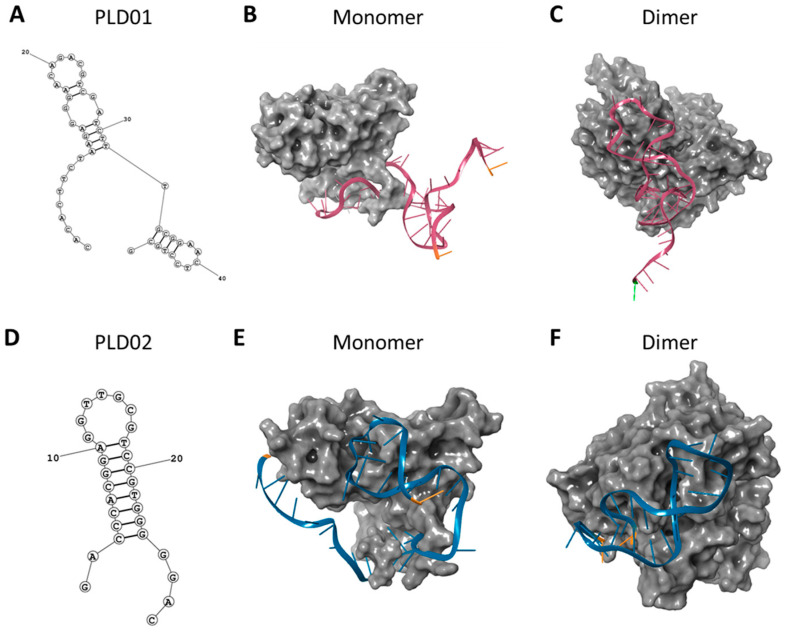
In silico docking of novel EpCAM-targeting aptamers to the EpCAM extracellular domain (EpEX). The 3D folded structures of (**A**) PLD01 and (**D**) PLD02 were generated using the Matthew’s group Fold Web Server. (**B**,**C**) PLD01 and (**E**,**F**) PLD02 were docked to the EpCAM extracellular domain (EpEX) monomer (**B**,**E**, PDB: 6i07) and *cis* homodimer (**C**,**F**, PDB: 4mzv) in Schrodinger maestro software.

**Figure 2:**
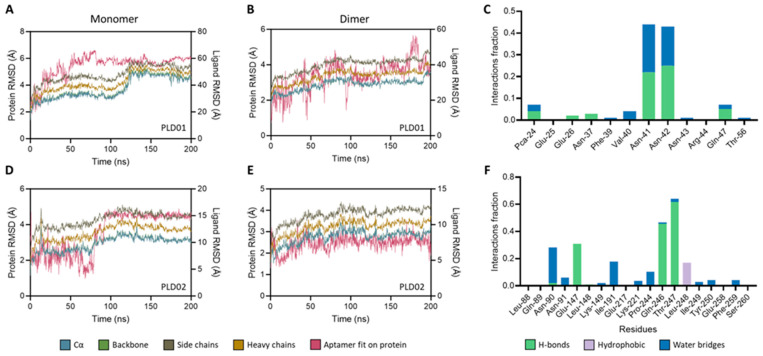
Characterization of the stability and binding of the novel aptamers to EpEX monomer and *cis* homodimer. Two hundred-nanosecond molecular dynamics (MD) simulations were performed with the epithelial cell adhesion molecule (EpCAM) ectodomain (EpEX) monomer or *cis* homodimer with (**A**,**B**) PLD01 or (**D**,**E**) PLD02. The resulting root mean square deviations (RMSD) plots with EpEX monomer–aptamer complexes and dimer–aptamer complexes were generated. The types of non-covalent interactions between the EpEX monomer and (**C**) PLD01 or (**F**) PLD02 during the MD simulations were identified.

PLD01 has a size of 47 nucleotides and possesses a stem-loop structure with two internal loops and single stranded segments at both termini (Figure 1A). PLD02 is 29 nucleotides and has a stem-loop structure with single-stranded segments at both termini (Figure 1D). PLD01 and PLD02 were docked to the EpEX monomer and *cis* homodimer, and these structures were used for MD simulations (Figure 1B,C and Figure 1E,F for PLD01 and PLD02, respectively). The PLD01-EpEX monomer complex was stable throughout the MD simulation, as indicated by maintaining a magnitude RMSD x ≤ 4 Å and achieving a highly stable conformation at ~100 ns (Figure 2A). A similar trend was observed for the PLD02-EpEX monomer complex (Figure 2D).

During the simulations, several residues on the EpEX monomer were identified at which the aptamer was predicted to make physical contact. These interactions were stabilized by non-covalent intermolecular interactions, and most notably with hydrogen bonds and water bridges (Figure 2C,F). The predicted interacting residues differed between the EpEX monomer and homodimer, emphasizing the importance of performing the simulations with both. Both the PLD01–EpEX homodimer and the PLD02–EpEX homodimer complexes achieved stable conformations during the MD simulations (Figure 2B,E). PLD02 made several contacts with the dimer residues and preferentially interacted with residues in the N terminal (NTD) and C terminal domains (CTD) of the EpEX dimer. The interactions with the NTD residues were also stabilized by non-covalent bonds.

**Figure 3:**
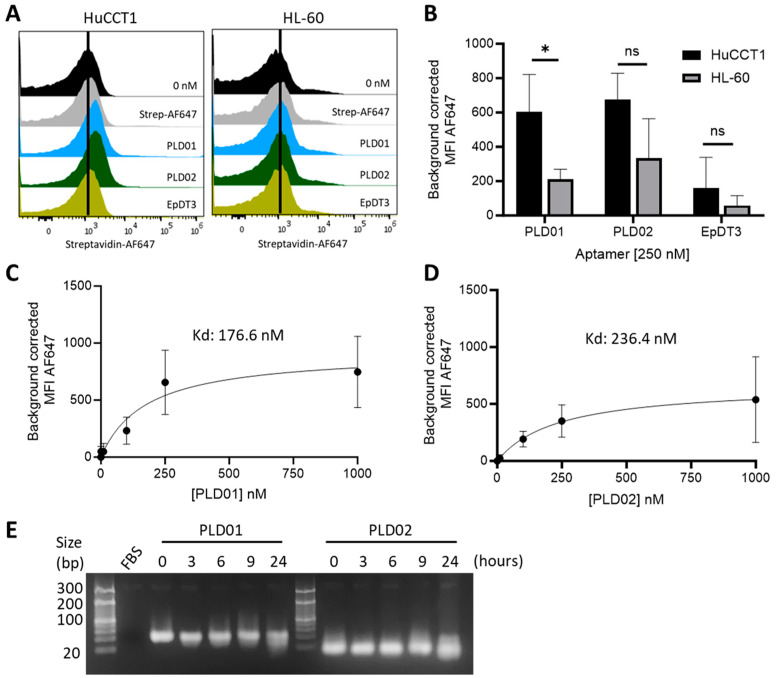
Binding specificity and affinity of aptamers to EpCAM^+^ cells. (**A**,**B**) The binding specificity of (250 nM) PLD01, PLD02, and EpDT3 biotinylated aptamers to EpCAM-positive HuCCT1 cells and EpCAM-negative HL-60 cells was evaluated using flow cytometry to quantify the mean fluorescence intensity (MFI) of streptavidin-AF647 on the cells. Cells were also stained with only streptavidin-AF647 to account for non-specific binding, and the MFI of these cells was used to perform background correction of the data. (**C**,**D**) HuCCT1 cells were incubated with different concentrations of (**C**) PLD01 or (**D**) PLD02 (0, 1, 10, 100, 250, or 1000 nM) and the binding affinity was evaluated using flow cytometry. A one-site specific binding curve was constructed to determine the dissociation constant (Kd) of each aptamer. All data represent the mean ± SD from three biological replicates. (**E**) PLD01 and PLD02 were incubated with 10% FBS at 37 °C for the time points shown. The samples were run on a 3% agarose gel and visualized using SmartGlow dye. (*) *p* < 0.05.

*Aptamers can bind to EpCAM+ cells*. The potential use of PLD01 and PLD02 for targeted delivery of therapeutic nanovesicles to EpCAM-expressing cells was evaluated. Cholangiocarcinoma (CCA) is a primary liver cancer with a TME that is characterized by an abundance of CSCs and a demonstrated correlation of EpCAM expression with survival [13,27,28]. To evaluate the aptamer binding specificity to EpCAM, we determined the binding of PLD01, PLD02, and EpDT3, an RNA aptamer that is known to target EpCAM, on either EpCAM-positive HuCCT1 CCA cells or EpCAM-negative HL60 leukemia cells. To assess the aptamer binding affinity, HuCCT1 cells were incubated with different concentrations of the aptamers. The aptamers exhibited preferential binding to HuCCT1 cells and had modest binding to HL-60 cells (Figure 3A,B). PLD01 and PLD02 both exhibited high binding affinities to HuCCT1 cells, with apparent Kd values of 176.6 nM and 236.4, respectively (Figure 3C,D). These findings demonstrated the affinity of the aptamers and their specificity for EpCAM-positive cells.

*Aptamers are stable in serum*. Given the susceptibility of DNA to nuclease-mediated degradation, the aptamers were synthesized with nuclease-protection modified bases. The serum stability of these aptamers was then assessed. However, there was minimal degradation of PLD01 or PLD02 observed during serum exposure at any time points evaluated (Figure 3E).

**Figure 4:**
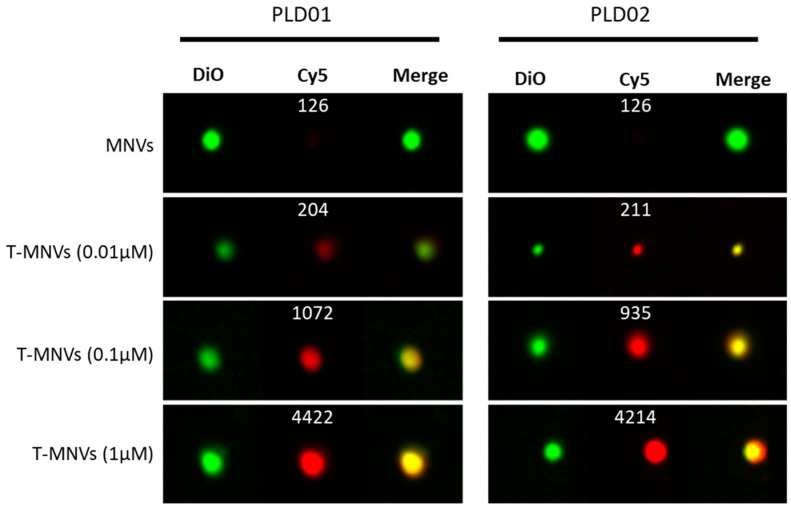
Decoration efficiency of milk-derived nanovesicles with novel DNA aptamers. DiO-stained milk derived nanovesicles (MNVs, green) were functionalized with 0, 0.01, 0.1, or 1 µM of Cy5-PLD01-TEG-cholesterol or CY5-PLD02-TEG-cholesterol (T-MNVs, red). The T-MNVs were evaluated using the ImageStream flow cytometer, and 10,000 events were acquired per sample. The Cy5 intensity was measured to quantify the decoration efficiency of PLD01 and PLD02 T-MNVs, and the average value is shown in each representative image in the Cy5 channel.

*Nanovesicles can be functionalized with aptamers*. Nanovesicle delivery vectors isolated from natural sources such as fat-free milk (MNVs) are an excellent delivery vehicle for therapeutic RNA molecules due to their low immunogenicity and the ability to be economically produced at a large scale [29]. Cholesterol-modified constructs can be incorporated into the MNV membrane due to the amphiphilic nature of their membrane. Using this approach, MNVs can be surface engineered and functionalized using aptamers for targeted delivery to the desired cells. The incorporation efficiency of 5′-Cy5-tagged, 3′-TEG-cholesterol-modified PLD01 and PLD02 aptamers into MNV membranes was evaluated using imaging-based flow cytometry. Aptamer decoration on MNVs was visible even at aptamer concentrations as low as 0.01 µM (Figure 4). Cy5 intensity on aptamer-functionalized MNVs increased in a dose-dependent manner proportional to the amount of aptamer used for functionalization (Figure 4, white values in Cy5 channel images). Both PLD01- and PLD02-functionalized MNVs showed similar signals indicating their effective incorporation into the membrane of MNVs.

**Figure 5:**
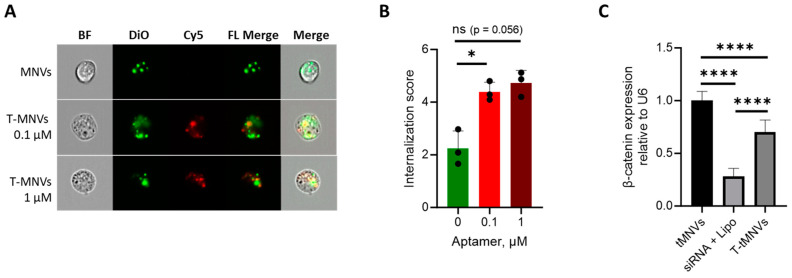
Evaluation of the uptake and bioactivity of EpCAM-targeting MNVs. HuCCT1 cells were treated with DiO-stained milk-derived nanovesicles (MNVs) functionalized with 0.1 or 1 µM of Cy5-PLD01-TEG-cholesterol aptamer. (**A**) The MNV uptake at 4 h post-treatment was visualized using the ImageStream system. (**B**) An internalization algorithm was run on the live, single, in-focus cell data. (**C**) MNVs loaded with β-catenin siRNA were functionalized with nothing (tMNVs) or 1 µM Cy5-PLD01-TEG-cholesterol (T-tMNVs). At 48 h post-treatment, β-catenin expression was measured using RT-qPCR. The data were normalized to tMNVs and expressed relative to U6. Cells transfected with β-catenin (siRNA + Lipo) were included as a positive control. The data represent the mean ± SD (n = 3 biological replicates). (*) *p* < 0.05, (****) *p* < 0.0001.

*Cellular uptake of aptamer-functionalized MNVs*. To evaluate their uptake efficiency by EpCAM-positive cells, MNVs were first functionalized with 0.1 µM or 1 µM Cy5-PLD01-TEG-Chol-modified aptamer or left unfunctionalized as plain controls. Nanovesicles were then incubated with HuCCT1 cells, and the rate of internalization was assessed after four hours (Figure 5A). Compared with the plain MNVs, there was a greater accumulation of aptamer-functionalized MNVs in EpCAM-positive HuCCT1 cells, as indicated by the MNV internalization scores (Figure 5B). However, the internalization rate was similar with MNV functionalized with either 0.1 µM or 1 µM PLD01. These findings demonstrate enhanced uptake of aptamer-functionalized MNVs by EpCAM-positive tumor cells.

*Efficacy of aptamer-functionalized MNVs containing siRNA*. Next, we assessed whether aptamer-functionalized nanovesicles could enhance the delivery of therapeutic cargo to EpCAM-expressing CCA cells. For these studies, siRNA against β-catenin was used as nuclear expression of β-catenin is associated with high-grade tumors, metastasis, and multidrug resistance in CCA cells, and this has been shown to be an effective RNA therapeutic for CCA [26,30]. MNVs were loaded with β-catenin siRNA and were kept unfunctionalized (plain tMNV) or functionalized with PLD01 to generate targeted therapeutic nanovesicles (T-tMNV). In comparison to plain tMNVs, treatment with PLD01 T-tMNVs induced greater silencing of β-catenin expression in HuCCT1 cells (Figure 5C). These findings show that MNVs functionalized with the EpCAM-binding aptamer PLD01 can be used to generate targeted therapeutic cargo delivery vehicles that retain the ability to deliver functional siRNA to EpCAM-expressing cells.

## 4. Discussion

Aptamer-based therapies have several advantages over conventional monoclonal antibody therapies such as superior solid tumor penetration and amenability to engineering. EpCAM is an attractive target for targeted therapies as it is highly expressed on cancer cells and has been implicated in tumor growth. Targeting cancer cells expressing EpCAM with specific aptamers could therefore enable the selective delivery of therapeutic agents. Using an *in silico* approach, we designed and screened two novel EpCAM-targeting DNA aptamers that could stably bind to both the EpEX monomer and the physiologically predominant *cis* homodimer. In vitro studies showed that both aptamers possessed high affinity toward EpCAM-positive CCA cells with modest binding to EpCAM-negative cells. These aptamers could be modified with functional groups and incorporated onto delivery vectors such as MNVs, while still retaining their EpCAM-targeting ability. The aptamer-functionalized MNVs can thus be utilized as vehicles to deliver therapeutic cargo in an EpCAM-targeted fashion.

The use of molecular docking and dynamics simulations to evaluate aptamer–target interactions and the stability of their ensuing complexes provides an efficient and cost-effective approach to develop new aptamers. Computational modeling further allowed for optimization of aptamer sequences based on predicted binding interactions. Future applications of machine learning will likely improve the ability to predict superior binding characteristics and enable rational design and thereby complement or supplant steps involved in traditional discovery approaches. In silico approaches for aptamer design further offer the ability to rapidly screen potential candidate sequences and predict their binding.

Several DNA and RNA aptamers that can bind to EpCAM have been developed, with dissociation constant (*Kd*) values that range from nanomolar to micromolar concentrations (41 nM–5.48 µM) [21,31,32,33]. Most of these, including the seed aptamers used herein for the *in silico* design, were identified using conventional discovery methods such as systematic evolution of ligands by exponential enrichment (SELEX). This method involves screening of a large oligonucleotide library pool, with PCR amplification of the positively selected candidates, followed by iterative rounds of screening [34]. While SELEX can yield the discovery of high affinity aptamers, it is time-consuming and expensive. The use of an *in silico* modeling approach to design and screen aptamer candidates that bind to a protein target of interest provides an alternative approach that has already led to the discovery of several high affinity EpCAM-targeting RNA aptamers [33,35]. In the study herein, PLD02 had sustained intermolecular interactions with residues in the TY loop domain/dimer interface region of the EpEX monomer, similar to the *in silico* findings between novel RNA aptamers and the EpEX monomer reported by Bell et al. [33]. Given that the homodimer is the predominant species, these residues might not be available for aptamer binding, thus highlighting the importance of performing modeling studies with both EpEX species. Interestingly, the aptamer performance *in silico*, namely the interaction dynamics with the EpEX dimer, did not directly reflect their bona fide binding affinity to EpCAM-positive cells in vitro. Whilst PLD01 exhibited the highest binding affinity in vitro, simulations did not produce strong affinity or contact with EpEX dimer residues.

The novel EpCAM-targeting aptamers described herein possess several positive attributes that support their potential for use in disease theranostics. Both PLD01 and PLD02 exhibited excellent serum stability, likely facilitated by the inclusion of phosphorothioate-modified bases within the aptamer sequences that conferred protection from endonucleases. Furthermore, the *in silico* modeling focused on EpCAM interaction sites via nucleotides within the loop regions of the aptamers. Thus, modifications at the terminal regions might have a lower impact on aptamer binding to EpCAM, and thus enable and support their conjugation to drugs or other constructs. Furthermore, the 3′ cholesterol modifications may have facilitated their persistence within cells. In addition to vesicle engineering, these attributes could support the use of these modified aptamers for intracellular delivery of therapeutic aptamer-siRNA chimeras via an encapsulation-free strategy [36]. A potential limitation of these aptamers as cell-targeting ligands is the possibility of eliciting on-target, off-tumor effects, given that EpCAM is expressed on healthy epithelial cells. Thus, high specificity for target antigens that are highly expressed in tumors is essential. Strategies to mitigate off-target effects can include the use of aptamers with modest binding affinities or with selective affinity to the post-translational modified version of the antigen uniquely found on tumor cells or by utilizing tumor microenvironment-sensitive aptamer protection mechanisms [37,38].

PLD01 and PLD02 were designed to generate EpCAM-targeted nanovesicle-based therapeutics. Cholesterol modified versions of these aptamers retained their EpCAM recognition and binding abilities and were successfully incorporated into the membrane of MNVs. MNVs as well as other biologically derived nanovesicles can be loaded with exogenous cargo, such as non-coding RNAs, and deliver this bioactive cargo to recipient cells [39]. MNVs were selected for this study due to their inexpensive and scalable production and favorable safety profiles in mice and zebrafish [29]. Although systemically administered MNVs exhibit liver-tropic distribution, active targeting to targeted cells of interest was desired to achieve the greatest therapeutic benefits. Indeed, whilst uptake studies showed internalization of MNVs by intrahepatic EpCAM-positive CCA cells, aptamer functionalized MNVs were internalized at a greater rate. Their increased uptake likely contributed to the enhanced functional effects observed in the cells treated with PLD01-functionalized therapeutic MNVs compared to plain therapeutic MNVs.

Despite successful use in pre-clinical models, the clinical translation of aptamer-directed therapies has been hampered and lags behind other oligonucleotide-based therapies [40,41]. Wider adoption of aptamer-based therapies will benefit from the availability of a broader repertoire of targeting aptamers of desirable specificity and binding affinity for tumor antigens.

## 5. Conclusions

An *in silico* approach was used to design novel EpCAM-targeting DNA aptamers based on an analyses of nucleic acid sequences that can interact with EpCAM. These aptamers have high binding affinity to and demonstrate specificity for EpCAM expressed on CCA tumor cells. Their binding abilities were not impeded by the addition of terminal moieties. These modified aptamers were effective targeting ligands and were able to direct the delivery of therapeutic nanovesicles to EpCAM-positive CCA cells. These aptamers may be useful for broader cell-targeting applications through conjugation with chemotherapeutic drugs, gene silencing constructs, peptides, or immune cell engagers, thus expanding the opportunities for EpCAM-targeted therapies.

## Figures and Tables

**Table 1:** Sequences of seed EpCAM binding oligonucleotides.

ID	Sequence 5′—3′	Sequence Length
JYK-01	5′-AGCAGCACAGAGGTCAGATGTGAAGGTT CGTTGTTTCGGTGGGTGTAGACTCTTTAGAAGAGATACA GATTTTGGGAATGCCTATGCGTGCTACCGTGAA-3′ [20]	100
Epp166	5′CGCGGAAGCGTGCTGGGCCAACAGAGGGACAAACGG GGGAAGATTTGACGTCGACGACACATAACCCAGAGGTC GAT-3′ [21]	77
SYL3C	5′-AGCGTCGAATACCACTACAGCACTACAGAGGTTGCGT CTGTCCCACGTTGTCATGGGGGGTTGGCCTGAGCGTCGA ATACCACTACAG-3′ [22]	88
Seq ID 63	5′-TGCGGCACACACTTCTATCTTTGCGGAACTCC TGCGGCTC-3′ [23]	40

**Table 2:** Oligonucleotide sequences of EpCAM-targeting aptamers used in this study.

Oligonucleotide ID	Sequence 5′—3′	Sequence Length
PLD01 (DNA)	5′-Biotin-C * A * C * ACTTCTAAGAGGG * AACAGACGTCGATCTTTGCGGAACT * CCTG * C * G-3′	47
PLD02 (DNA)	5′-Biotin-G * A * C * CCACGGAGGTTG * CGTCCGTG* GGGG * A * C-3′	29
EpDT3 (RNA)	5′-Biotin-GCGACUGGUUACCCGGUCG-3′	19
Cy5-PLD01-TEGChol	Cy5-C * A * C * ACTTCTAAGAGGG * AACAGACGTCGATCTTTGCGGAACT * CCTG * C * G- TEG-Cholesterol	47
Cy5-PLD02-TEGChol	Cy5-G * A * C * CCACGGAGGTTG * CGTCCGTG * GG GG * A * C-TEG-Cholesterol	29

(*) indicates phosphorothioate-modified bases. TEG, triethylene glycol.

## Data Availability

The raw data supporting the conclusions of this article will be made available by the authors on request.

## Abbreviations

B-Aptamers, biotinylated aptamers; CCA, cholangiocarcinoma; CSC, cancer stem cells; EpCAM, epithelial cell adhesion molecule; EpEX, EpCAM ectodomain; *K_d_*, dissociation constant; MD, molecular dynamics; MNVs, milk-derived nanovesicles; MNV-NC, non-target control siRNA-loaded MNVs; ND, N terminal domain of EpEX; RMSD, root mean square deviation; RMSF, root mean square fluctuation; SELEX, systematic evolution of ligands by exponential enrichment; TAA, tumor-associated antigen; TEG-Chol, triethylene glycol cholesteryl; TME, tumor microenvironment; T-MNV, EpCAM aptamer-targeted MNVs; tMNVs, therapeutic PD-L1 siRNA-loaded MNVs; TY, thyroglobulin domain; 3WJ, three-way junction RNA.
